# Multimodal Non-Destructive In Situ Observation of Crystallinity Changes in High-Density Polyethylene Samples with Relation to Optical Parameters during Tensile Deformation

**DOI:** 10.3390/s24196367

**Published:** 2024-09-30

**Authors:** Karoline Felbermayer, Sandrine van Frank, Bettina Heise, Markus Brandstetter, Christian Rankl, Harald Ladner, Peter Burgholzer

**Affiliations:** 1 RECENDT—Research Center for Non Destructive Testing GmbH, Science Park 2, 2.OG, Altenbergerstrasse 69, 4040 Linz, Austria; sandrine.vanfrank@recendt.at (S.v.F.); bettina.heise@recendt.at (B.H.); markus.brandstetter@recendt.at (M.B.); peter.burgholzer@recendt.at (P.B.); 2 TCKT—Transfercenter für Kunststofftechnik GmbH, Franz-Fritsch-Straße 11, 4600 Wels, Austria; harald.ladner@tckt.at

**Keywords:** crystallinity, HDPE, optical parameters, terahertz spectroscopy, optical coherence tomography, infrared spectroscopy, Raman spectroscopy

## Abstract

Many non-destructive optical testing methods are currently used for material research, providing various information about material parameters. At RECENDT, a multimodal experimental setup has been designed that combines terahertz (THz) spectroscopy, optical coherence tomography (OCT), infrared (IR), and Raman spectroscopy with a tensile test stage. This setup aims to gather material information such as crystallinity and optical parameters of high-density polyethylene (HDPE) during a tensile test. The setup compares common IR and Raman spectroscopy and the less common optical methods THz and OCT. Complementarity is achieved through different frequency ranges and measurement approaches, resulting in different measured optical material parameters and depths. During tensile testing, HDPE samples with varying crystallinity were analysed, and the determined optical parameters such as refractive index, birefringence, scattering coefficient of decay, and penetration depth can be correlated with the change in crystallinity. These findings demonstrate that the optical methods and their outcomes can be interconnected. With further optimization of the experimental setup, it would be possible to observe the alignment of fibres in fibre composite panels and the stress distribution of polymers effectively. This opens interesting possibilities for polymer characterization in the future, including quality control during moulding processes and material testing.

## 1. Introduction

Optical methods offer a wide range of non-destructive solutions for the material characterisation of polymers. A correlation of data from polarization-sensitive terahertz time domain spectroscopy (PS-THz TDS), optical coherence tomography (OCT) and Raman infrared (IR) spectroscopy with results from in situ tensile testing offers the opportunity to illustrate the relation between crystallinity and optical parameters [1,2,3].

Crystallisation is a transformation process which is observed in thermoplastic polymers (e.g., HDPE) and occurs during solidification of a melt or a solution: a non-ordered phase changes into an ordered and denser phase. In this process, it passes through two states: the nucleation and the growth state. The orientation, amount and size of crystals are important for the physical properties of polymers and are controlled by the temperature gradient [4,5]. Different thermo-mechanical treatments can result in different degrees of crystallinity, meaning different weight percentages of crystalline compared to the amorphous part of a polymer.

The crystallinity of a semi-crystalline polymer is an important characterization factor and has an impact on the stiffness, strength, toughness properties and optical properties [2,6]. In practice, routine transformations of polymeric materials such as moulding require the application of shear or deformation and heat. The result is a product with different microstructures in different locations, and the mechanical properties depend on the microstructure [7]. Tracking structural changes, in particular crystallinity, during a tensile test allows to better understand and quantitatively estimate the degree of crystallinity change when deforming a polymer in practical use cases [4,6,8,9]. It should be noted that various structural changes occur during the tensile deformation of semi-crystalline polymers, e.g., undrawn samples with an isotropic spherulitic structure collapse during yielding and drawn samples form a fibrillary structure [10,11].

The most widely used method to estimate crystallinity is density thermal analysis differential scanning calorimetry (DSC), in which the specific enthalpy of fusion of a partially crystalline sample is compared to one a similar sample with 100% crystallinity would have. Other conventional methods which allow for time-resolved crystallinity measurements are X-ray diffraction (XRD), nuclear magnetic resonance (NMR), and infrared spectroscopic methods (such as Attenuated Total Reflectance Fourier Transform Infrared (ATR FTIR) and Raman IR spectroscopy) [5,9,12].

In this paper, we explore two alternatives to the conventional methods for estimating changes in crystallinity, namely optical coherence tomography and polarization-sensitive terahertz time-domain spectroscopy (PS-THz-TDS). These methods present the advantage of being able to measure in situ, therefore following the impact of stress in real time and in a realistic environment without affecting the material. Due to the long wavelength of radiation, they also give information about sub-surfaces or bulk parameters. They are indirect methods in that they measure variation in parameters such as birefringence and turbidity, which are consequences of stress-induced crystallization [2]. Therefore, for the experiments presented here, IR spectroscopic methods such as ATR FTIR- and Raman spectroscopy were kept as reference methods [10,13].

THz time domain spectroscopy (THz-TDS) is a relatively new but promising technique for the characterization of materials, especially polymers [14,15,16,17,18,19]. OCT is well-established in the biomedical application field, but also in the field of material testing of polymers, especially for the detection of defects and inner structural changes [20,21,22,23]. It allows to observe the change in thickness, optical penetration depth and scattering factor of the sample during the mechanical tests. These parameters are also influenced by the change in crystallinity and correlate with it [24,25].

## 2. Methods, Materials and Experimental Setup

### 2.1. Methods

#### 2.1.1. Estimation of the Crystallinity with Standard Methods

The most common methods to estimate the crystallinity of polymers are DSC, XRD, FTIR and Raman spectroscopy [9]. Zerbi et al. [26] proposed this empirical relation to estimate the crystallinity X_C_ with FTIR:

(1) $$X_{C}=\left(1-\frac{\frac{1-\frac{I_{A}}{I_{C}}}{1.233}}{1+\frac{I_{A}}{I_{C}}}\right)\;*\;100\%$$

X_C_ is the percentage of the crystalline part; I_A_ and I_C_ are the intensities of the absorption at the characteristic bands. There are two characteristic bands at wavenumbers 730 cm^−1^ and 1474 cm^−1^ for the amorphous phase and at wavenumbers 720 cm^−1^ and 1464 cm^−1^ for the crystalline phase of the HDPE. The bands at the wavenumbers 1474 cm^−1^ and 1464 cm^−1^ will be used later for calculation. The ratio factor 1.233 is the relation for the fully crystalline HDPE from the literature [13,27,28].

Raman spectroscopy and NMR are the only two techniques which allow for a direct evaluation of the three phases of crystallinity of polymers (crystalline, interphase and amorph). By observing vibration bands in the wavenumber region 900–1500 cm^−1^, Raman spectroscopy provides a quantitative determination of the three crystallinity phases [29]. As an internal standard to estimate the crystallinity, the CH_2_ twisting modes of the crystallinity chain (1298 cm^−1^) and the amorphous chain (1313 cm^−1^) in the spectral range of 1250 to 1350 cm^−1^ are used. Therefore, a curve-fitting program (pseudo-Voigt function) is used [10,12,30].

(2) $$X_{C}=\left(1-\frac{I_{1313}}{I_{1298}}\right)\;*\;100\%$$

#### 2.1.2. Estimation of Crystallinity from THz Data

**Figure 1:**
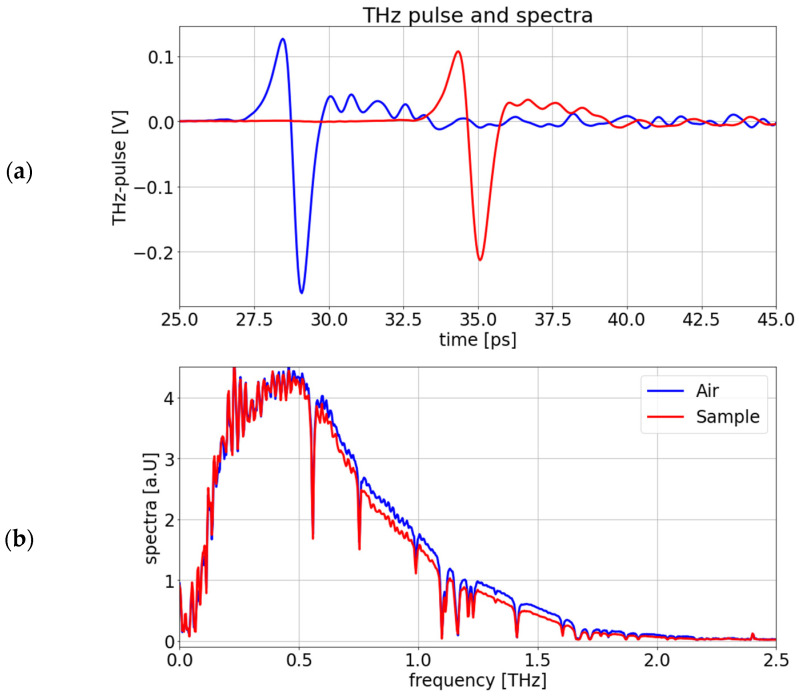
(**a**) THz pulse measured in air as reference (blue) and through an HDPE sample (red) in transmission with a spot size of 1 cm. (**b**) The fast Fourier-transformed THz spectra of the air reference pulse (blue) and the sample pulse (red).

We use a time-domain spectrometer emitting a short THz pulse to probe the samples. The THz pulses are then transformed in a THz spectrum by fast Fourier transformation (FFT). For the determination of parameters, a reference THz pulse in air is measured, as shown in Figure 1.

The differences in amplitude and phase of the signal with respect to the reference are directly related to the absorption coefficient α(f) and refractive index n(f) of the sample. The complex refractive index $\underline{n}=n+i\kappa$ consists of the real part n and the imaginary part κ:

(3) $$n(f)=1+\frac{c_{0}}{\omega *d}*\Delta \phi (f)$$

(4) $$\kappa \left(f\right)=\frac{\alpha \left(f\right)*c_{0}}{\omega }$$

where d is the sample thickness, $c_{0}=2.99792458\;*\;10^{8}\frac{m}{s}$ is the speed of light in vacuum, ω=2 ∗ π ∗ f is the radial frequency and $∆\phi \left(f\right)=\phi_{sample}\left(f\right)-\phi_{reference}(f)$ descripts the phase difference.

By taking the ratio of the phase measured along two perpendicular directions of propagation, we can estimate a “birefringence” parameter independent of the sample’s thickness:

(5) $$∆n(f)=\frac{n_{0{}^{\circ}}(f)-1}{n_{90{}^{\circ}}(f)-1}=\frac{{\Delta \Phi }_{0{}^{\circ}}(f)}{{\Delta \Phi }_{90{}^{\circ}}(f)}$$

As the refractive index of HDPE is expected to be almost constant in the frequency range achievable with the THz setup [14], the data were effectively averaged within the range 0.7–2 THz, i.e., the mean of the refractive indices at 0° and 90° linear polarization over this range is used to calculate the birefringence as defined above.

For the determination of crystallinity from polarization-sensitive THz-TDS data, it is important to extract several optical parameters from the raw THz-TDS data, such as the birefringence, the refractive index of the sample and the absorption constant within the THz spectral range [8,31].

The density of the crystalline phase is higher than the density of the amorphous phase. Thus, the crystallinity X_C_ can also be determined according to its different densities [8].

The crystallinity in the weight percentage of HDPE (X_cHDPE_) with density ρ can be estimated with the values of the densities of 100% amorphous (ρ_a_ = 0.85 g/cm^3^) and 100% crystalline (ρ_c_ = 1 g/cm^3^) phases, as reported by Lin et al. [12]:

(6) $$X_{c}=\left(\frac{\rho_{c}}{\rho }\right)*\frac{\rho -\rho_{a}}{\rho_{c}-\rho_{a}}*100\%$$

(7) XcHDPE=ρ−0.85gcm30.15∗ρ∗100%

The relation of the density with the THz data is based on the Lorentz–Lorenz equation [8]:

(8) $$\frac{\underline{n^{2}}-1}{\underline{n^{2}}+2}=\frac{\rho *\underline{a}}{3*\epsilon_{0}}\Rightarrow \rho =\frac{\underline{n^{2}}-1}{\underline{n^{2}}+2}*\frac{3*\epsilon_{0}}{\underline{a}}$$

with complex refractive index $\underline{n}=n+i\kappa$; complex polarization is $\underline{a}$ (very small for HDPE), and the permittivity of vacuum is $\epsilon_{0}=8.8542\;*\;10^{-12}\frac{As}{Vm}$. This implies a correlation between the change in crystallinity and the THz data. It also provides the opportunity for direct calculation of crystallinity, which is intended for future data analysis.

A change in crystallinity during a tensile test is expected to affect the birefringence of the material in the THz domain due to the formation of crystals with molecular chains oriented perpendicular to the elongation axis [11].

#### 2.1.3. Estimation of Crystallinity from OCT Data

The extraction of optical properties from OCT data is well established in the biomedical field [24,25], but also in the material field [22,23,32,33]. An in situ investigation of the flow-induced crystallisation of polymers was observed and analysed in [34]. With OCT, it is possible to perform a depth-resolved analysis because the imaging is based on the back-reflection from the turbid polymer.

**Figure 2:**
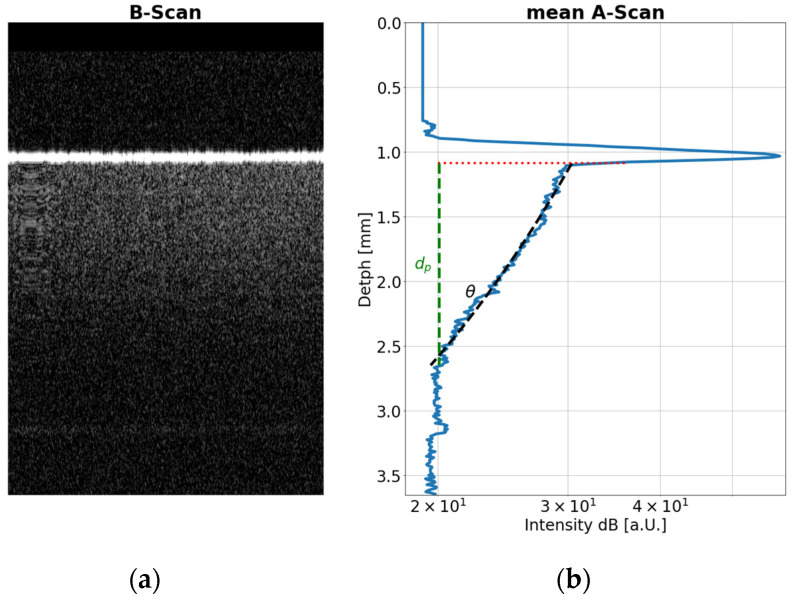
(**a**) The B-scan of an HDPE sample over a lateral scan range of 4 mm and a depth of 3.6 mm. (**b**) The mean A-scan averaged over the lateral scan range in the B-scan. It shows the penetration depth d_p_ (green line) at 1 dB level (+Offset) and the scattering decay coefficient θ (black line), here the 10 dB decay.

For this study, the cross-sectional images (B-scans) of the centre of the samples were observed during the tensile testing. An important result of these measurements was the determination of the thickness change, which is needed in the multimodal data analysis in combination with THz sensing to calculate the refractive index change. Another necessary step is the characterization of the scattering layer thickness (d_p_) and the decay coefficient (θ) in the media from the mean depth profile (A-scan), as shown in Figure 2 [25,35].

### 2.2. Materials

High-density polyethylene (HDPE) is a commonly used thermoplastic semi-crystalline polymer with a simple linear structure and few branches. Unbranched polyethylene can easily crystallize, which causes high hardness, high stiffness, high cohesion and high density. The typical crystallinity of HDPE is between 50 and 80% [31].

For this study, three types of samples with different attempted degrees of crystallisation were produced by injection moulding at the TCKT in Wels. Plates with the same injection moulding cycle of 73 s and a size of 170 × 170 mm^2^ with a thickness of 4 mm were moulded. The HDPE was pre-dried for three hours at a temperature of 90 °C. Different crystallinities of the HDPE plates were achieved by applying different tool temperatures of 15 °C, 40 °C and 70 °C, as mentioned in Table 1.

**Figure 3:**
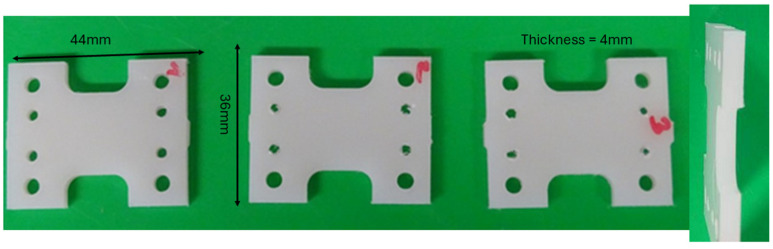
Photos of the punched samples for the tensile test from the front side (**left** picture) and from the cross-section (**right** picture). The numbers of samples, 1 to 3, define the different crystallisation states of the samples (1: low, 2: standard and 3: high crystallisation). The form of the sample was designed to fit perfectly in the tensile stage.

For the measurements, a couple of samples of each type were punched from the plates, as shown in Figure 3. The design of the sample was determined in such a way that it fits perfectly into the tensile stage.

During a standard tensile test, HDPE shows a homogenous deformation up to the first yield point, then the stress decreases to a second yield point. After the second yield point, an inhomogeneous deformation follows, which ends in a necking. After the necking, the stress increases again and continues in a strain-hardening region [10]. In this study, only the first part, the homogeneous deformation, was observed for stress-induced crystallisation and a correlation was found with the optical parameters of the methods used.

### 2.3. Multimodal Experimental Setup (MMS)

**Figure 4:**
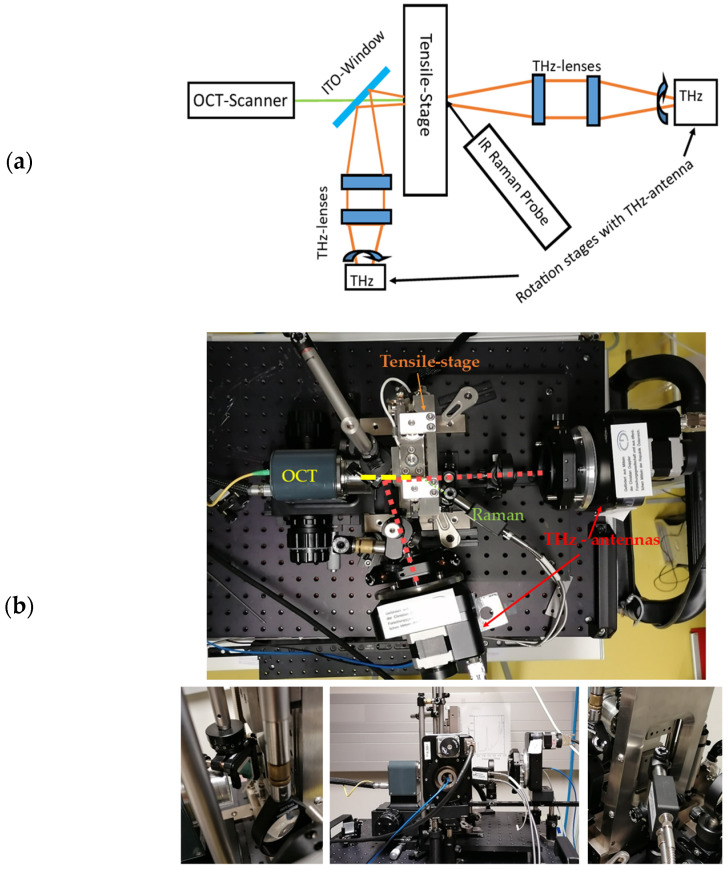
(**a**) Schematic drawing of the multimodal experimental setup. The OCT laser beam is through an ITO (indium tin oxide glass) window focused on the sample from the left side. The Raman setup is measured from the right side. Also, the THz radiation is focused from the left side of the sample and deflected at the ITO window to the THz antenna. (**b**) Photo of the multimodal experimental setup, containing Raman, OCT and THz configuration, from different viewpoints.

The centre of the MMS is a tensile stage where the samples are fixed for the tests. The stage is surrounded by a combination of three optical NDT technologies: THz time domain spectroscopy (THz-TDS), infrared Raman spectroscopy (IR-Raman) and optical coherence tomography (OCT), which are shown in Figure 4.

The applied OCT system is a commercial swept-source OCT (Thorlabs VEGA System), with a centre wavelength of 1300 nm, a spectral range of 100 nm and a lateral resolution of 20 µm. Because of the high imaging depth of 12 mm of the OCT system, the whole cross-section of the sample could be imaged. It provides information about the sample thickness changes as well as the change in absorption depth and the change in optical properties (e.g., reflectance, scattering decay coefficient and the penetration depth) of the samples.

The THz time domain spectroscopy (TDS) system comprises a pulsed fibre-based femtosecond laser (Menlo Tera K15) and two photoconductive antennas: an emitter and a receiver. These antennas are mounted on custom-made motorized stages to allow for rotation with 1-degree accuracy. Both the emitted and detected THz radiation have linear polarization, enabling straightforward determination of birefringence. This is achieved by rotating the antennas to 0° and 90° positions. The beam size at focus is about 1 cm.

The employed Raman spectroscopy system is a highly sensitive and high-resolution fibre optic Raman system from Wasatch Photonics with a 785 nm excitation laser. The system covers the spectral range of 92–3396 cm^−1^ and offers a resolution of 8 cm^−1^. The beam diameter is about 1.5 mm. As mentioned above, Raman spectroscopy is well suited for the determination of the crystallisation properties and is often used as a standard method for this purpose [10,12,30].

From the geometry of the setup, one sees that the Raman measurement and the OCT measurements are necessarily carried out each from one of the surfaces of the sample, while the THz measurement is carried out in transmission. The light spot for each apparatus has been focussed as precisely as possible on the centre of the sample.

The in situ tensile stage is a dual leadscrew 5 kN tensile testing stage from Deben. It is a flexible stage which allows for reflection and transmission measurements. The jaw extension is measured using a capacitive extensometer with a resolution of 300 nm and the motor speed is regulated with optical rotary encoders fitted to the motor. The force is monitored using a standard strain gauge load cell. The strain rates go up from 5 µm/min to 4 mm/min.

In the experiment, the sample was stretched to a length of 3.5 mm at a strain rate of 0.5 mm/min. This was due to the high tensile modulus of HDPE and the uniform deformation at the beginning of the stress–strain behaviour. After reaching the desired length, the stretching process was stopped and held at this position for the rest of the measurement period. In total, each measurement takes a maximum of one hour.

## 3. Results and Discussion

**Figure 5:**
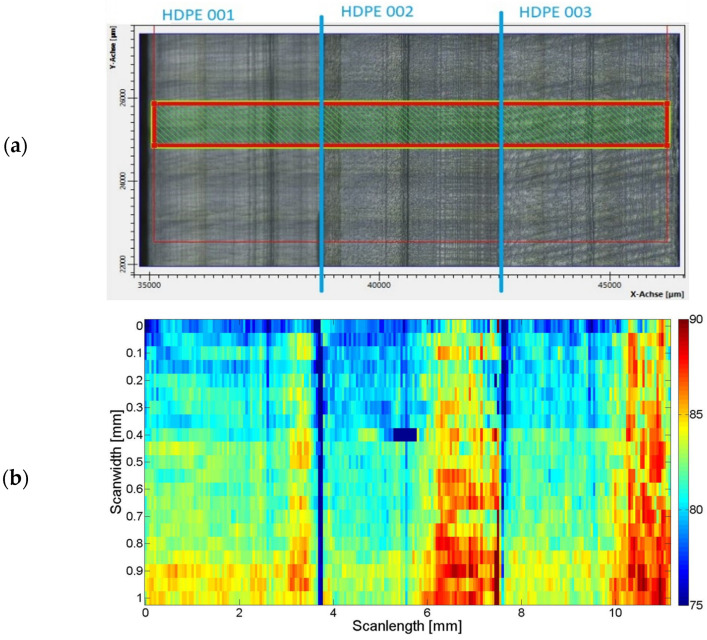
(**a**) Photo of the cross-sectional 3 jointed HDPE samples with the FTIR microscope scanning area marked as red box. (**b**) The calculated crystallinity over the cross-sections from ATR FTIR spectra bands I_A_ (1464 cm^−1^) and I_C_ (1474 cm^−1^) are based on Equation (1) [13].

An estimation of the sample crystallinity (by the Lumos ATR-FTI microscope, Bruker Optik GmbH, Ettlingen, Germany) at different positions and over the cross-section, i.e., the thickness, of the samples was conducted prior to tensile testing. It revealed that for all samples, the crystallinity varies over the cross-section. This is an effect of the injection moulding process, during which the cooling rate of the material varied depending on the position in the mould, hence the inhomogeneous crystallinity (Figure 5).

While the *low*-crystallinity sample HDPE001 exhibits on average a lower degree of crystallization, the *standard*- and *high*-crystallinity samples are very similar. This denomination is therefore not used anymore in the rest of the text.

**Figure 6:**
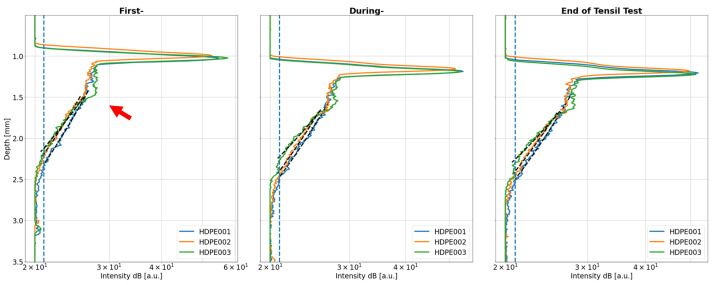
OCT mean A-scans of the HDPE samples 001–003 with plateau (red arrow), recorded at the start (First-), mid (During-), and end of the tensile test. The blue vertical line shows the 1 dB level and the black dashed line shows the decay parameter.

The crystallisation effect during moulding was observed in [34] and confirmed by a polarization-sensitive OCT setting similar to the one used in this study. The effect is observable as a plateau similar to the one we observe (see the red arrow) in all our OCT A-scans taken at the start, during and at the end of the tensile test measurements, as shown in Figure 6. Here, the plateau after the surface peak of HDPE003 is significantly more distinct than at the A-scans of the other samples. A difference in the plateaus of HDPE001 and HDPE002 is hard to find. From the result of the cross-section measurements, one would expect either all three samples to exhibit the same behaviour (measurement from the side of lowest crystallinity) or for HDPE002 to exhibit a plateau similar to HDPE003 (measurement from the side of highest crystallinity). The reason for the plateau shown by HDPE003 is that it was positioned with the side of highest crystallinity turned toward the OCT device, while by HDPE001 and HDPE002, the side of lowest crystallinity was measured.

**Figure 7:**
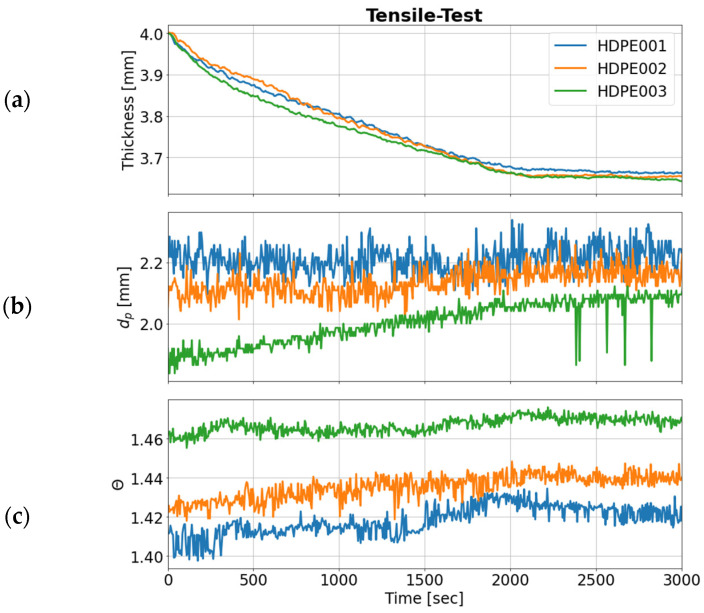
Comparison of the features extracted by OCT imaging over time: change in (**a**) the thickness, (**b**) penetration depth, and (**c**) scattering coefficient of decay of the OCT mean A-scans of HDPE samples (HDPE001—blue line, HDPE002—orange line, HDPE003—green line) during the tensile test.

As mentioned above, HPDE has a high tensile modulus and homogenous deformation to a defined point. The direct comparison of the OCT extracted features during the tensile test is given in Figure 7, also showing similar results for HDPE001 and HDPE002 (measurement on the side with low crystallinity) and a marked difference for HDPE003.

**Figure 8:**
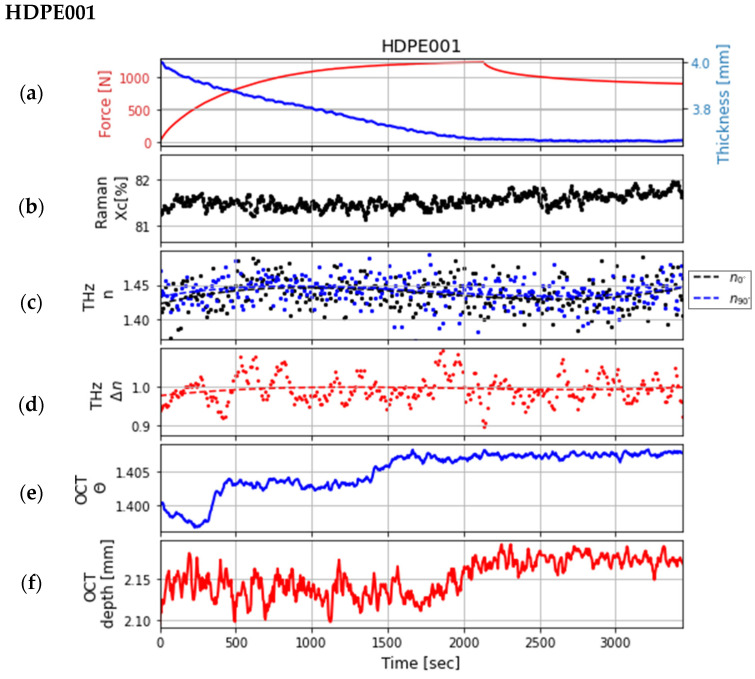
Comparison of the extracted features of the sample *HDPE001* recorded over time: (**a**) the measured force at the tensile stage and the change in the sample thickness; (**b**) crystallinity X_C_ [%] estimated with Raman spectroscopy; (**c**,**d**) refractive indices at 0° (black dots) and 90° (blue dots) and the birefringence (red dots) estimated with polarization-sensitive THz measurements, each fitted with a polynomial regression (corresponding dashed lines); (**e**,**f**) are both parameters determined from OCT data: decay parameter θ and penetration depth $d_{p}$.

**Figure 9:**
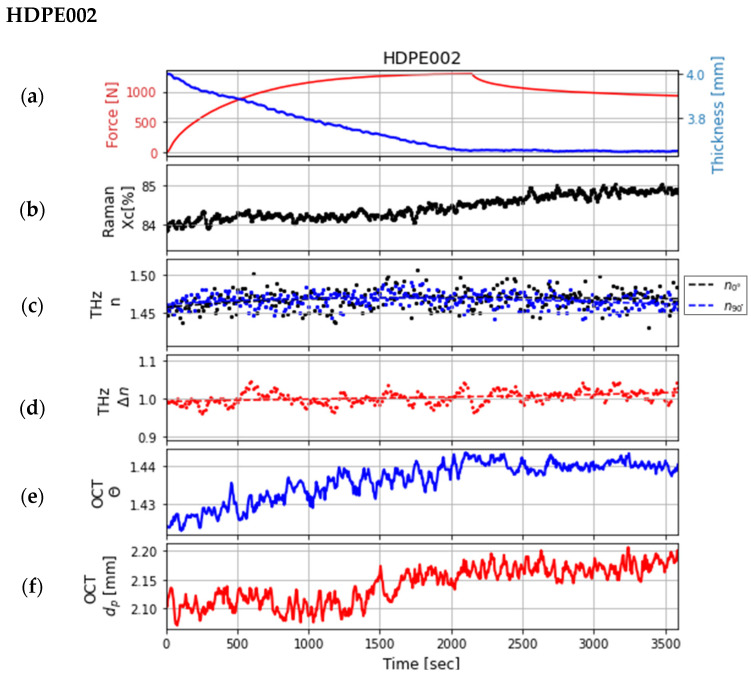
Comparison of the extracted features of the sample *HDPE002* recorded over time: (**a**) the force measured at the tensile stage and the change in the sample thickness; (**b**) crystallinity X_C_ [%] estimated with Raman spectroscopy; (**c**,**d**) refractive indices at 0° (black dots) and 90° (blue dots) and the birefringence (red dots) estimated with polarization-sensitive THz measurements, each fitted with a polynomial regression (corresponding dashed lines); (**e**,**f**) are both parameters determined from OCT data: decay parameter θ and penetration depth $d_{p}$.

**Figure 10:**
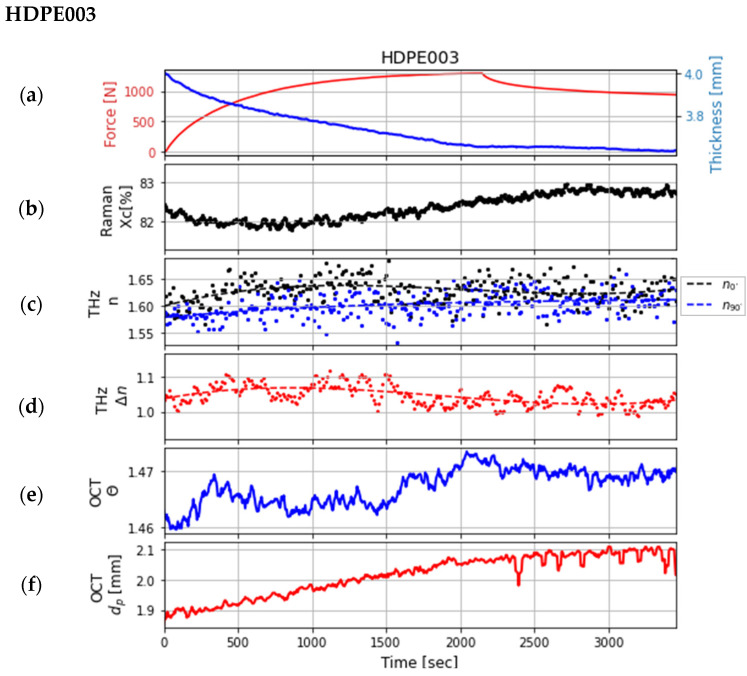
Comparison of the extracted features of the sample *HDPE003* recorded over time: (**a**) the force measured at the tensile stage and the change in the sample thickness; (**b**) crystallinity X_C_ [%] estimated with Raman spectroscopy; (**c**,**d**) refractive indices at 0° (black dots) and 90° (blue dots) and the birefringence (red dots) estimated with polarization-sensitive THz measurements, each fitted with a polynomial regression (corresponding dashed lines); (**e**,**f**) are both parameters determined from OCT data: decay parameter θ and penetration depth $d_{p}$.

Synchronizing the multimodal system was a challenging but essential step. For this study, the data of the three samples from measurements of one day were used after a perfect synchronization. The comparison of the properties obtained using the different technologies is illustrated in the following three figures (Figure 8, Figure 9 and Figure 10). Each graph (a) in the three figures depicts the change in the force during the tensile test, which was measured at the tensile stage, and the change in the thickness of the sample, which was estimated with the OCT system. Each graph (b) shows the change in crystallinity at a single point of the sample, which was estimated using the data from the Raman system at the bands at 1298 and 1313 cm^−1^. These bands were chosen because they do not shift during deformation [10]. The change in the refractive indices and the birefringence calculated from the data of the polarization-sensitive THz system (after smoothing the data using polynomial regression) are shown in graphs (c) and (d). The refractive indices are in the range as described in the literature [14,19]. Graphs (e) and (f) show the extracted features (penetration depth and scattering coefficient of decay) from the data measured by the OCT system.

In Figure 8, the graphs of the sample HDPE001 are depicted. The graphs illustrate the changes during the tensile test; only a very slight oscillation can be seen in the change in crystallinity at the sample’s surface determined from the Raman data. However, at the end of the tensile tests, the surface crystallinity is slightly higher than at the beginning. A slight oscillation is also visible in the change in THz refractive indices and birefringence after smoothing the data. This variation remains however within the noise of the measurement. The extracted features from the OCT data show significant variations. The impact of the tensile test on the crystallinity and the extracted features of the sample is thus low but visible in the Raman and OCT data.

In Figure 9, the graphs with the sample HDPE002 are depicted. The change in the crystallinity during the tensile test is higher compared to the sample with low crystallinity. Furthermore, a slight oscillation in the change in the refractive indices in the THz data can be noted; however, it is still within the measurement noise. When smoothing the data, the change in the birefringence exhibits a slight slope, which could be linked to changes in the bulk of the polymer plate. As with the first sample, the changes in the penetration depth and decay parameter extracted from OCT data are greater during the tensile phase and remain almost stable during the relaxation phase. These changes are small.

The tensile test results for the sample HDPE003 are illustrated in the graphs of Figure 10. It appears that the crystallinity from the Raman data is lower than for HDPE002. This is due, as previously mentioned, to the sample HDPE003 being inadvertently placed the other way round (i.e., with the side of lowest crystallinity toward the Raman sensor). Overall, the crystallinity from the Raman measurement increases slightly during the test. This change in crystallinity is visible and is inversely reflected in the altered THz refractive indices and their change in birefringence. The penetration depth varies much more over time than in the case of the other two samples with lower crystallinity. The scattering decay parameter determined from the OCT data is also much higher, but the change over time during tensile testing is similar.

All three samples show a variation in surface crystallinity as determined by Raman spectroscopy during tensile testing. Changes in the bulk as measured via THz TDS were small and within the measurement noise, such that variations can be inferred from the smoothed data but must be confirmed by future measurements. A direct correlation between the change in crystallinity during the tensile test and the penetration depth, as well as the scattering coefficient of decay, cannot be found, but the penetration depth becomes higher during the tensile test and the decay parameter becomes steeper. In general, the penetration depth is higher at low crystallinity and the decay parameter is shallower than at high crystallinity. The fact that the penetration depth and scattering coefficient of decay become higher over the tensile test is probably due to the change in orientation and compression of the crystals due to the tensile test.

## 4. Conclusions and Outlook

The study proposes a measurement approach combining multiple optical methods for non-destructive material testing that allows for correlating material information, potentially leading to better characterization. The Raman measurements on the three samples with different crystallinities showed that, as expected, crystallinity changes with tension, also exhibiting correlations with the data from the two other methods. The connection with the variation in the refractive indices in the THz range, on the one hand, can at this point only be guessed in the smoothed data, while the effect on the penetration depth and the scattering decay coefficient, on the other hand, is clear in the raw OCT data. For better material characterization, a THz-TDS system with a larger bandwidth could be used, with which variations in the absorption peak expected at 2.3 THz for HDPE could be analysed. Moreover, a polarization-sensitive OCT system could be used instead of the swept-source OCT system, which gives more information about the stress distribution in the samples and its influence on crystallinity. The optimization and synchronization of different systems, along with their data analysis utilizing AI and machine learning, offer further potential for material characterization and production process monitoring.

## Figures and Tables

**Table 1:** Samples and their different tool temperatures and crystallinities.

Name	Tool Temperature [°C]	Crystallinity
HDPE 001	15	Low
HDPE 002	40	Standard
HDPE 003	70	High

## Data Availability

Research data are stored in an institutional repository and will be shared upon request to the corresponding author.
